# Which preferences associate with school performance?—Lessons from an exploratory study with university students

**DOI:** 10.1371/journal.pone.0190163

**Published:** 2018-02-16

**Authors:** Daniel Horn, Hubert Janos Kiss

**Affiliations:** 1 Institute of Economics, Centre for Economic and Regional Studies of the Hungarian Academy of Sciences, Budapest, Hungary; 2 Department of Economics, Faculty of Social Sciences, Eotvos Lorand University, Budapest, Hungary; Middlesex University, UNITED KINGDOM

## Abstract

Success in life is determined to a large extent by school performance so it is important to understand the effect of the factors that influence it. In this exploratory study, in addition to cognitive abilities, we attempt to link measures of preferences with outcomes of school performance. We measured in an incentivized way risk, time, social and competitive preferences and cognitive abilities of university students to look for associations between these measures and two important academic outcome measures: exam results and GPA. We find consistently that cognitive abilities (proxied by the Cognitive Reflection Test) are very well correlated with school performance. Regarding non-cognitive skills, we report suggestive evidence for many of our measured preferences. We used two alternative measures of time preference: patience and present bias. Present bias explains exam grades better, while patience explains GPA relatively better. Both measures of time preferences have a non-linear relation to school performance. Competitiveness matters, as students, who opt for a more competitive payment scheme in our experimental task have a higher average GPA. We observe also that risk-averse students perform a little better than more risk-tolerant students. That makes sense in case of multiple choice exams, because more risk-tolerant students may want to try to pass the exam less prepared, as the possibility of passing an exam just by chance is not zero. Finally, we have also detected that cooperative preferences—the amount of money offered in a public good game—associates strongly with GPA in a non-linear way. Students who offered around half of their possible amounts had significantly higher GPAs than those, who offered none or all their money.

## Introduction

There is growing literature that indicates that individual preferences studied by economists affect and predict a wide range of choices made at the individual level. For example, risky choices like smoking, drinking, not having insurance, holding stocks rather than Treasury bills or choosing an occupation with a high earning risks are positively and significantly correlated with risk attitudes (see for instance [1,2,3]. Similarly, time discounting predicts behaviour in many walks of life like health (e.g., BMI—see for instance [4] or creditworthiness—see [5]; savings—see [6]; or credit card balance—see [7]), labour supply and lifetime income [8,9,10]. However, note that the relationships are not always unambiguous, for example for time discounting and health [11,12]. Moreover, in many cases not all measures related to a preference have a predictive power, only some of them [13,14].

In this exploratory study, we focus on educational performance. Using experimental tasks in a university classroom, we attempt to see which preferences may affect school performance. Understanding the factors that shape school performance is of utmost importance as school performance determines to a large extent the success in life as captured, for instance, by the wage premium [15] or the positive relation between schooling and other socioeconomic outcomes (e.g., health—see for instance [16], or voting—see [17]). We consider both cognitive and non-cognitive skills (preferences) and try to measure some of them in the classroom. Note that cognitive and non-cognitive skills are not orthogonal abilities as for instance cognition affects many aspects of human behaviour [18,19]. There is a growing literature that shows that *both* cognitive ability and non-cognitive skills help to predict outcomes in life besides school performance. Examples include workplace performance, marital status, and risky behaviours [18,20,21,22,23,24,25,26,27].

Then we relate the measures obtained to two outcomes of observable school performance of bachelor students: the exam result of a subject (Economics) and the grade point average (henceforth, GPA) of the semester when the exam was taken. More precisely, we study four interrelated preferences that have received considerable academic attention in the last decades: risk, time, social and competitive preferences. These areas are interrelated as any temporal choice involves risk as the future is inherently uncertain [28]. Therefore, measures of these preferences may be correlated, as we show later. Why is it important to consider the association between these preferences and school performance? We know that academic success depends positively and to a large extent on intellectual ability. Borghans et al. [18] report that IQ predicts outcomes in several fields of life (e.g., job performance and longevity) and is the best predictor for two academic outcomes (college grades and years of education) when compared to the Big Five personality factors. While the importance of cognitive abilities to explain academic success is a general finding, many studies also point out that they rarely explain more than 50% of the variance in academic performance [29,30]. In fact, Borghans and colleagues [31] report much smaller, below 0.1, R^2^-statistics of IQ on wages using the British Cohort Study or the National Longitudinal Survey of Youth ‘79. Duckworth and Seligman [32] show in a longitudinal study that self-control explained more than twice as much variance in the final grades of eighth-grade students as IQ. In fact, the part not explained by cognitive skills is perhaps best understood considering personality traits and preferences. Some remarks are in order. First, Eckstein and Wolpin [33] study the association of preferences on high school dropouts, but they consider different preferences (as preference for leisure, consumption value of school attendance) and show that those who drop out have different traits than those who graduate. Second, we note that gender differences have been documented extensively regarding these preferences [34,35]. However, since our subject pool is not balanced regarding gender, we do not focus on this aspect. Third, there are many other potential factors that affect school performance. For instance, Stinebrickner and Stinebrickner [36] show the harming impact of working during school on academic performance.

We report here briefly our main results. In line with the existing literature, we find that cognitive abilities are important both for exam results and GPA. We consider two aspects of time preferences, patience, and present bias, and they affect exam results and GPA in an expected way. Patience is non-linear as it matters only for the very impatient. Also, future-biased and time-consistent students perform similarly well, but better than the present-biased student. The more present-biased a student is, the worse her expected grades are. Risk preferences seem to affect the exam result in the following way: the more risk-averse a student, the better are the exam results. Competitive preferences are weakly related to exam results and GPA in an expected way. That is, the more competitive a student is, the higher is her grade. Related to competitiveness, we find also that more overconfident students do worse in the exams but not in GPA. We find an interesting non-linear effect of social preferences: compared to those who contribute nothing/everything to a public good, those who contributed half of their endowment, fared significantly better regarding their GPA. A caveat on social preferences is in order. Our analysis is carried out on an individual level, while cooperativeness may be important on the group or class level. Hence, classes that exhibit a higher degree of cooperativeness may be more successful academically, ceteris paribus. If this is the case, then social preferences should be measured and compared on the group level. Note that the effects that we document are often non-linear.

The differential effect of the preferences on exam results and GPA may be due to the different nature of these two. The exam that we use to measure school performance was an Economics exam for students not enrolled in the Economics program. Possibly, students do not consider it a major or important subject in their track, even though it has a weight in the GPA that may be important, for example, if they want to enter a Master program. On the other hand, GPA encompasses all subjects, giving a more balanced view of school performance. Moreover, the exam was a multiple-choice test with clear good answers; hence the exam grade can be seen as an objective measure. However, in the GPA there may be subjects that are not evaluated on such objective scales. For example, oral exams or written assignments in the form of an essay seem to let subjectivity play a larger role in grading; hence, one may expect here that non-cognitive skills are more important.

The rest of the paper is structured as follows. Next, we review the preferences that we study and their potential connections to educational performance. We also formulate some hypotheses about how these preferences may affect school performance. Then in section 3 we present the experimental tasks that we use and validate our measurement of the preferences. Section 4 contains the results that consider both linear and non-linear relationships. In section 5 we discuss the results and outline some possible venues for future research.

## Literature review and hypotheses

### Time preference

Time preferences are important as many decisions involve costs and benefits that occur at different points in time. Given the costs and efforts to achieve a goal, many individuals find hard to delay the benefits. For instance, the marshmallow tests showed that the ability to delay gratification is positively related to many desirable behaviours and outcomes (for instance, better health, higher average SAT points, more rewarding social relationships, see [37,38]).

Following the literature, we consider two aspects of time preference: patience and present bias. Considering patience and present bias together is known as the (*β*, *δ*) formulation, see for instance Burks et al. [39]. The choice between different amounts of money at different points in time may reveal the implied discount factor that characterizes an individual. The more an individual values future payoffs, the more patient she is and probably the more she is willing to sacrifice to get those future rewards. When the discount factor is measured on different horizons, but involving the same time interval (say, now vs. 1 week later and in a year vs. in a year and a week), then the difference in discount rates on the different horizons is also informative. If an individual is more impatient on the short-term horizon than on the long-term, then she exhibits *present bias* that may make it hard for the individual to make efforts immediately, and she tends to procrastinate efforts and costs. Other studies relate these concepts to willpower and self-control. Several studies (see for example the survey by Bucciol et al., [40] and the references therein) note that self-control and willpower are limited resources and can be exhausted, a process described by psychologist as ego depletion.

How do time preferences affect educational outcomes? It is natural to think that those students who are more patient and hence value the future more and/or who are abler to delay gratification (that is, make efforts and study instead of playing or hanging around), will be academically more successful. There is ample empirical evidence in this regard. Golsteyn et al. [9] use longitudinal data from Sweden that links measured time preference at age 13 to later life outcomes gained from administrative registers. Their results indicate that high discount rates are related to worse school performance, among others. More patient individuals had significantly higher grades throughout the school years and were more likely to attain a university diploma. They also show that the effect operates through early human capital investments. Importantly, the authors control for a host of potential confounds. For instance, there is some evidence that time preferences and cognitive abilities are related [3,19], but cognitive abilities are controlled for in the study. Cadena and Keys [41] report very similar findings in a US setting, and Falk et al. [6] also show in a world-wide survey that patience is significantly related to educational attainment. In a Dutch setting, Non and Tempelaar [42] find that impatience is associated with lower exam performance, but not with less effort. De Paola and Gioia [43] use data from a sample of Italian undergraduate students and document a negative relationship between individual discount rates and school performance. Burks et al. [44] also report significant relationship in the expected direction between time preferences and academic performance at university (timely graduation and final GPA), while Beattie et al. [45] also point out that college divers are more likely to procrastinate than college thivers. Sutter et al. [38] find evidence that children and adolescents in Austria who are more impatient show worse conduct during school years (smoke more and drink more alcohol). Castillo et al. [46] document similar findings in the US. However, the empirical findings are not unambiguous as Bettinger and Slonim [47] fail to find any correlation between patience and school performance.

Similarly to the above-cited studies, in our corresponding experimental task, we used intertemporal monetary choices (also known as “money earlier or later” (MEL) decisions, see [48]) to measure individual patience of the participants over two horizons. Consequently, we can measure both patience and present bias.

Hypothesis 1 (Time preference): Based on the literature we conjecture that patience has a positive and present bias may have a negative effect on school performance.

### Risk preferences

Even though we speak only about risk preferences, recent research has revealed that risk and uncertainty (or ambiguity)–though related—are different concepts. Broadly speaking, risk preferences capture how much individuals value safe and certain outcomes relative to risky (when probabilities of different events are known) or uncertain (when those probabilities are unknown) alternatives.

There is some evidence that risk preferences are related to schooling, but the direction of the effect is unclear. For instance, Guiso and Paiella [49] show that more educated people are more risk-tolerant, but we do not know if schooling makes individual less risk-averse or risk aversion affects schooling decisions. Relatedly, Brañas-Garza et al. [50] and Brañas-Garza et al. [51] find no relationship between math skills and risk attitudes. Sutter et al. [38] find that experimental measures of risk and uncertainty aversion predict field behaviour (but not performance) in the school only weakly. Analyzing an Italian panel data set Belzil and Leonardi [52] find that risk attitudes only modestly explain whether an individual is admitted to higher education or not. Burks et al. [44] find a weak negative association between risk aversion and GPA among university students, but the correlation vanishes as controls are added to the model. Hartlaub and Schneider [53] discuss how risk aversion and social background affect school choice and show compelling evidence in favour of the class-specific effect of risk aversion in Germany.

We note that risk and time preferences may be correlated. For instance, Leigh [54] reports a significant negative correlation between time discount rates and risk aversion. Anderhub et al. [55] find a statistically significant negative correlation between risk aversion and discount factors in a within-subject design. However, being correlated does not mean that they are the same. In an experiment, Coble and Lusk [56] reject the hypothesis that risk, and time preferences are governed by a single parameter and conclude that the relationship between the two is that individuals prefer to delay the resolution of risk. Andreoni and Sprenger [29] also state that while risk and time preferences are intertwined, they are not different manifestations of the same phenomenon. Epper and Fehr-Duda [57] show that risk attitudes and time discounting are related through various channels, for example both risk tolerance and patience are on average higher for payoffs that materialize in the future compared to payoffs in the present. Epper and Fehr-Duda [57] offer a unifying framework to capture all the interactions. In this study, we do not investigate the mechanisms that relate time and risk preferences, and they are not significantly correlated according to our data.

Hypothesis 2 (Risk preference): Based on the literature we do not expect a strong effect of risk attitudes on school performance.

### Social preferences

Social preferences express the idea that other individuals’ utility also enters the utility function of the decision-maker. It includes many aspects of human behaviour ranging from trust and reciprocity to cooperation. Social preferences received a lot of attention form researchers lately and many aspects and associations have been studied (e.g. the relationship between the body mass index and social preferences, see [58]).

It seems plausible that in a more trusting and cooperative environment students help each other improving educational attainment in general. There is scant evidence that social preferences and especially prosocial behaviour are related to school performance. Using Italian data, Caprara et al. [59] find that prosocial attitude measured at age 8 predicted educational performance and peer acceptance 5 years later. This study did not use experimental tasks, but assessment reported by the children themselves and teachers. Layous et al. [60] also report that prosociality is related to peer acceptance, suggesting a potential link between prosocial attitudes, peer effects and educational attainment. We are not aware of any paper that uses experimental tasks to relate prosocial behaviour to school performance.

We focus on cooperation and measure it with a two-person variant of the public goods game. Some studies show that choice in the public goods game is positively related to effort in the field, see for instance Englmaier and Gebhardt [61] or Galizzi and Navarro-Martinez [62]. That is why we conjecture that larger contribution in this game may imply more effort in studying that eventually results in better performance, ceteris paribus.

Hypothesis 3 (Cooperativeness): We expect that cooperativeness goes well with higher school performance.

### Competitive preferences

Competitive preferences may be important in school performance as individuals that are competitive may want to excel at school as well. The only study that we are aware of in this regard is Azmat and Iriberri [63] who find such results in a natural field experiment during which students in a high school in Spain received for a year information that allowed them to know if they performed above or below the class average and the distance from the average. The provision of this information increased students’ grades by 5%.

Hypothesis 4 (Competitiveness): Based on Azmat and Iriberri [63] we expect that more competitive participants may have better results in the exam, ceteris paribus.

### The experimental tasks

There were 3 occasions when we measured preferences using experimental tasks, all of them carried out during university lectures. In total 242 students (144 women, 82 men, 16 missing) participated in the occasions. The Disciplinary and Ethics Board of the Faculty checked our design and stated that there are no ethical problems with the experimental tasks. When approving the research, they also asked to guarantee that the participation is voluntary and that we deal with the data with due confidentiality. Moreover, they demanded that completing the experimental tasks did not affect in any way the grade of the students in the given course. At the beginning of the sessions we explained that participation is voluntary and anybody who does not want to participate could leave the room and return when it ends or remain seated and do something else (without disturbing the participants). We asked if everybody understood that participation was voluntary and when we saw that there were no questions, we took it as a verbal consent. We did not obtain a written consent for several reasons. First, the Disciplinary and Ethics Board of our Faculty did not ask for it, but was satisfied if we obtained the verbal consent by explaining thoroughly that participation was voluntary. Second, as the experimental tasks were completed during lectures and we used the time of a professor, we tried to make things as swiftly as possible. Handing out and then recollecting written consents would have taken several minutes and would have lengthened the exercise. Subjects were undergraduate students enrolled in different programs of the Eötvös Lóránd University (Budapest, Hungary). No student participated in more than one session. The first/second / third session was carried out on 17. November 2015 / 27. November 2015 / 3. December 2015 with students mainly enrolled in International Affairs and Computer Science programs. Note that there is a potential sample bias in our investigation as most of the students are enrolled in International Affairs and Computer Science. More research is needed to find out if this is the case and to which extent are our results representative. Each session lasted about 40–45 minutes.

We had a paper-and-pencil implementation of the experimental tasks. After seating the participants, they received the instruction sheets that also contained the situations in which the participants had to decide. At the beginning of each occasion, an experimenter read aloud the relevant information that the participants could also follow using the instruction sheets. For the exact instructions, see S1 Appendix or https://dx.doi.org/10.17504/protocols.io.kepctdn. More precisely, we made clear that participation was voluntary and anonymous. We also explained that we wished to connect answers in the experimental tasks to school performance of the subject, so we asked the participants to provide their code of the electronic education administration system. Providing the code was also voluntary, and we pointed out that after connecting the data we would erase the code to make identification impossible. Next, we explained that the experimental tasks would consist of six independent decisions and that after completing the experimental tasks we would select two participants randomly who would be paid according to their decisions in one of the randomly chosen decisions. The instruction sheets were numbered and each had two additional tags with the number of the sheet. When handing in the sheets, participants kept one of this number tags and the other was used to select the two participants to be paid. We also made clear that some of the payments related to some of the decisions may be postponed to 1, 2 or 3 weeks later and explained carefully how they would receive the money in these cases. After all these explanations the experimenter answered the questions that emerged.

Participants made the decisions in the six situations using the instruction sheet that explained the scenarios with examples (see S1 Appendix). The order of decisions was the same for all subjects that raise potential concerns regarding order effects (see more on order effects in [64]). As the number of our subjects is relatively small compared to the number of experimental tasks, we could not deal with this problem by randomizing the order of the tasks properly. Note also that as there was no feedback between tasks, the order effect (if present) probably is a slight one.

The first decision measured cooperation with a two-person public goods game. We explained on the instruction sheet that the participants would be randomly matched with another participant and both start with an endowment of 4000 Ft (12.7 EUR / 14.1 USD) and could contribute any amount of this endowment to a common account, without knowing the contribution of the co-player. Each of them would receive 70% of the total contributions to the common account, independently of the individual contribution. The final payoff is the sum of the money from the common account plus the endowment that has not been used for contribution. We also made clear that if after completing the experimental tasks this situation is chosen for the payoff, then we would pick another participant randomly and pay according to her decision. Note that optimal decision from an individual point of view is to contribute zero to the common account, as a unit contribution returns only 0.7 that is the marginal benefit is less than the marginal cost. However, from a societal point of view contributing all the endowment is the optimal decision as a unit contribution generates 2*0.7>1 units. We consider the contribution to the common account as a natural measure of cooperation: the more a participant contributes, the more cooperative she is.

The second decision meant to gauge the risk attitude of the participants. Similarly to Sutter et al. [38], we elicited risk (and uncertainty) attitudes using the Ellsberg two-color choice task [65]. We told the participants that a bag contained 10 black and 10 red balls and we would draw one ball from the bag. Each participant was endowed with 3000 Ft (9.5 EUR/ 10.6 USD) and could choose the color to bet on and the amount to bet on the color of the ball drawn. We explained that if the participant guesses correctly the color of the ball, then we would double the bet. We consider the amount of the bet as a natural measure of risk aversion: the less a participant bets, the more risk-averse she is. In the analysis we will use the amount not placed on the bet as a measure of risk aversion. We also explained that if this decision is chosen for payoffs, then we would again pick randomly two participants, carry out the drawing of the ball and pay according to their decisions.

The third decision was similar to the previous one, but we wanted to capture uncertainty aversion. Therefore, in this case the distribution of the balls in the bag was unknown to the participants. Again, they were endowed with 3000 Ft (9.5 EUR/ 10.6 USD) and could choose the color to bet on and the amount to bet on the color of the ball drawn. The payoffs were as before: we doubled the bet if the the bet was correct. Randomly allocated, either after decision 3 or 5 there was an optional extra task. It represented a maze and we put clearly that it is not necessary to solve this exercise, but those who do it successfully would receive an extra 300 Forints (0.95 EUR / 1.1 USD) if chosen to be paid at the end of the experimental session. The idea of including the maze was to see if ego depletion affects the completion of this task. We found no effect and since it is not tightly related to the rest of the paper, we ignore this task henceforth.

Decision 4 and 5 tested time preferences. More concretely, we used choices from two multiple price lists in which participants were asked to make a series of decisions between a smaller reward (Forint X) in period t and a larger reward (Forint Y > = Forint X) in period τ>t. We kept Forint Y constant and varied Forint X in t. In decision 4 we asked participants if they preferred 2400 Forints (7.6 Eur / 8.5 USD), 2500 Forints, 2600 Forints and so on up to 3500 Forints (11.1 EUR / 12.4 USD) today instead of 3500 Forints in a week. Based on previous experimental evidence we expected that in the first decision participants would choose the later, but substantially larger payoff, while in the last decisions they would prefer the earlier payoff. Based on the switching point in between these two we can calculate a proxy of their individual discount rate or patience over this horizon (now vs. 1 week later). Note that it is only a proxy as the indifference point between the 3500 Ft to be received in a week and the money immediately is between the switching point and the previous choice. Thus, if somebody prefers 3500 Ft in a week to 3200 Ft now, but then chooses 3300 Ft now instead of 3500 Ft in a week, then her indifference point is between 3200 and 3300 Ft. The closer is the switching point to 3500 Forints, the smaller is the individual discount rate / the more patient is the student. Decision 5 was identical, but there the earlier / later decision referred to amounts to be received in two / three weeks. In this decision we can also use the switching point to calculate the individual discount rate for each participant. Individual discount rates over these horizons are interesting *per se*, as previous work has shown that the degree of patience predicts behaviour [4,6,9,10,14]. Moreover, the relative magnitude of these patience measures reveals if somebody is present-biased or not. If the individual discount rate in decision 4 is larger, than that in decision 5, then the individual is more impatient over the short run, than over the long run and suffers present bias. Present bias may affect many decisions through procrastination. Several studies indicate that students tend to procrastinate in their academic tasks, see for instance Solomon and Rothblum [66], Steel [67] or experimental evidence [68,69]. There is a wide range of real-world phenomena affected by present bias, for example credit-card borrowing [14], saving decisions [70,71] or physical exercise [72] to name a few. Present bias implies that the individual has difficulties to delay gratification and this—as explained before—may affect academic achievement. Those that exhibit the reverse relationship are called future-biased. Future bias implies to delay taking a reward, a strange phenomenon at first sight. It has received scarce attention, see Loewenstein [73], Rubinstein [74] or Sayman and Öncüler [75]. We measure present bias as the difference of the switching points on the two horizons (switching point at later horizon minus switching point at earlier horizon). Therefore, if a participant switches from the 3500 Ft in a week to the earlier payoff at 2800 Ft, but then on the later horizon she switches at 3000 Ft, then her present bias measure is 200 Ft. Burks et al. [39] compare the predictive power of several time preference measures and show that using discount rates and present bias together predicts best outcomes.

Three remarks are in order. First, in other studies these time preference tests involve a farther away time horizon for the second set of questions. For instance, Meier and Sprenger [14] use now vs. 1 week and 6 weeks vs. 7 weeks, Dean and Ortoleva [76] use now vs. 1 week and 5/6 weeks vs. 6/7 weeks. However, in our case, we had to opt for a shorter timeframe as Christmas break was approaching which could have jeopardized the payment (had it been selected). Second, we explained carefully that if the payment involved getting paid later, then the participants would receive the corresponding amount of money in an envelope from the teacher of the course that we used to carry out the experimental tasks. We also added that if there was any problem with the payment, they should contact the secretary of the Department of Economics. Third, we had the time preference tasks close together. Individuals attempting to be consistent may have remembered their switching point in decision 4 and make the same choice in decision 5. Hence, possibly, we underestimate present bias.

After decision 5 we had an 8-item test (see S1 Appendix for the exact questions). The first three questions were a variant of the Cognitive Reflection Test [77]. We reformulated the questions and used different numbers to minimize the possibility that somebody remembered the questions and the right answers from some earlier experience. The rest of the questions involved general and popular knowledge (e.g., How many verses are there in an elegiac couplet?; How many seasons did the series Friends have?). We asked the participants how many of the questions they thought they answered correctly. They could have received 250 Forints plus if they guessed correctly. The difference between this guess and the actual number of correct answers is a measure of overconfidence. The test served as a basis for decision 6 in which participants had to decide how they would like to be paid for their correct answers in the test. They could choose a flat-rate compensation of 250 Forints (0.79 EUR / 0.88 USD) per correct answer (maximum amount to be received this way is 2000 Forints (6.3 EUR / 7.1 USD) or a competitive compensation that consisted in picking randomly two other participants and compare the number of correct answers. If the participant to be paid had more / an equal number of / less correct answers than the better one of the two randomly chosen students, then she would receive 4500 / 2500 / 0 Forints (14.3 / 7.9 / 0 EUR 15.9 / 8.8 / 0 USD). The choice of the second compensation scheme reveals competitive preferences in a binary way. Note that if a subject is unsure in the answers to the questions, then she may prefer to stay away from the competition, so there may be a downward bias in this measure. A more neutral task (e.g. one in line with Niederle and Vesterlund, [78]) would have been more adequate to measure competitiveness but would have been more difficult to implement. We consider that even given the potential downward bias, we capture those subjects who are really competitive.

The number of correct answers to the first three questions measures cognitive skills. Several studies [77,79,80] report a statistically significant correlation of about 0.4 between CRT performance and other tests of analytic thinking (e.g., ACT, NFC, SAT, and WPT). There are other ways to measure cognitive abilities (for instance, the Raven test), but there are substantially longer. Moreover, the fact that we could embed the CRT into the quiz is another advantage. However, we should note that CRT is somewhat mathematical in content and may not capture every facet of the cognitive skills that affect the GPA and exam results of our subjects. Note also that CRT is not only about cognitive capacity, but also measures disposition for judgement and decision making, and is a good predictor of strategic behaviour and associates with social preferences as well (see more on CRT in [81]).

After the 6 decisions, we gathered some additional information and asked the gender, the year of birth and the highest level of education of the participant.

After everybody handed in the instruction sheets with the answers, we rolled a die to determine which of the 6 decisions determines the payoffs and picked two participants randomly. We looked their corresponding decisions and paid on the spot. The random choice of the payoff-relevant decision never was one of the time preference tasks (decision 4 and 5) that may have involved delayed payment.

### Measurement validation and descriptive statistics of the preferences

Our main objective is to see which preferences correlate with school performance and we can draw strong conclusions only if we can validate our preference measures. Table 1 below summarizes our main measures.

**Table 1:** Descriptive statistics of our main variables.

Variable	Full sample					With valid exam or GPA scores				
	Obs	Mean	Std. Dev.	Min	Max	Obs	Mean	Std. Dev.	Min	Max
Time now (HUF)	198	273	252	0	1100	150	285	252	0	1100
Time later (HUF)	198	295	265	0	1100	150	319	271	0	1100
Time now (discount factor)	198	0.92	0.07	0.69	1	150	0.92	0.07	0.69	1
Time later (discount factor)	198	0.92	0.08	0.69	1	150	0.91	0.08	0.69	1
Risk	242	1451	787	0	3000	183	1382	759	0	3000
Uncertanity	241	1807	806	0	3000	182	1746	801	0	3000
Present Bias	198	22	226	-1100	700	150	35	236	-1100	700
Cooperativeness	239	2430	1241	0	4000	180	2499	1179	0	4000
Competitiveness	241	0.56	0.5	0	1	183	0.58	0.49	0	1
Cognitive (CRT)	242	1.36	1.04	0	3	183	1.14	1.04	0	4
Cognitive (knowledge)	242	1.14	1.01	0	4	183	1.25	1.02	0	3
Female	226	0.64	0.48	0	1	169	0.70	0.46	0	1
Exam	149	2.95	1.26	1	5	149	2.95	1.26	1	5
GPA	154	4.05	0.46	2.81	4.95	154	4.05	0.46	2.81	4.95

Altogether we have more than 200 observations, but only have exam or GPA grades for around 150. We kept the variables unstandardized, which makes interpretation easier. However, for some variables, we have divided the number by 100 (for cooperativeness, time and risk preferences) so that the coefficients are more easily readable.

Time now / later represent our time preference measures on the shorter (now vs. 1 week) and longer (2 vs. 3 weeks) horizons. The mean indicates that relative to the 3500 Ft to be received at the later date when do they switch to the earlier amount. Hence, 273 in Time now shows that on average our subjects’ indifference point is 3500–273 = 3227. We also compute the corresponding discount factors that are in line with those found in the literature, see for instance Frederick et al. [82]. Risk and uncertainty show the attitude toward uncertainty as the amount that the participants *not* placed on the bet. Present bias, as explained above, measures the difference in the switching points of the two time-horizons. Here we present the difference that can be either positive or negative. However, remember that the definition of present bias is that somebody is more impatient now than later, so for these individuals the present bias measure is positive. Those with a negative measure are future-biased, while time consistent individuals have a present bias of zero. In other studies about one-third of the participants are found to be present biased [14,83], and in our case, it is very similar: 30 per cent.

Cooperativeness is gauged as the contribution to the common project in the public goods game that may range from 0 to 4000. In public goods game, this contribution usually amounts to 40–60 per cent of the initial endowment [84]. The mean in our experimental task is at the upper end of this range.

Competitiveness is a binary measure that is 1 for those who chose the competitive compensation scheme after the quiz and is 0 for those who opted for a piece rate payment. Compared to Niederle [35], a survey that deals extensively with competitiveness, the 56 per cent in our sample is in line with the usual numbers reported in the literature.

As explained already, we measured cognitive abilities using the Cognitive Reflection Test. Our measure Cognitive (CRT) reports the mean of the correct answers. Our mean of 1.36 correct answers is within the usual range, and according to Frederick [77], it places our sample between Harvard University (mean of 1.43) and University of Michigan (mean of 1.18). Remember that our quiz consisted of 8 questions, and the first three were those of the Cognitive Reflection Test, while the rest were general knowledge questions. Cognitive (knowledge) indicates the average number of correct answers to these questions; Table 1 indicates that on average students could only answer 1.14 of the five questions correctly. We use this measure to capture abilities or knowledge not explained by the Cognitive Reflection Test.

Female shows that 64 percent of the participants were females. Exam and GPA are our performance measures. Grades in an exam may range from 1 to 5, 5 being the best grade. Since the GPA is an average of the exam grades, in principle, it also ranges from 1 to 5, but as shown by the data the actual range is more compressed.

In S1 Appendix we report the survey in full. In S2 Appendix, we present to some detail the study by Dean and Ortoleva [76], who have recent findings on the relationship between most of our variables within one experiment. We are not aware of other experimental studies that investigate all these measures within one experimental session. Table 2 reports the pairwise correlations between Dean and Ortoleva’s [76] measures and Table 3 shows the correlation between our measures. Dean and Ortoleva do not report the correlations between the last three variables (see Table 3 in Dean and Ortoleva [76]). We represent all correlations and signal the ones that are significant at 1 / 5 / 10% levels.

**Table 2:** Correlation coefficients and significance level between variables in Dean and Ortoleva [76].

	Time Now	Time Later	Risk	Uncertainty	Trust Sender	Trust Return
Time Now	1					
Time Later	0.67, ***	1				
Risk	0.32, ***	0.15	1			
Uncertainty	0.18, **	0.17, **	0.46, ***	1		
Trust Sender	-0.02	-0.09	-0.04	-0.04	1	
Trust Return	0.02	-0.04	0.07	0.1	0.46, ***	1
Cognitive	-0.08	-0.23, ***	-0.06	-0.11	0.1	-0.01
Overconfidence	-0.02	-0.01	0.04	0.05	0.06	0.09
Female	0.06	0.11	-0.07	-0.11	-0.02	0.08

** p<0.05,

*** p<0.01

**Table 3:** Pairwise correlation between our variables.

	Time now	Time later	Present bias	Risk aversion	Uncertainty aversion	Cooperativeness	Competitiveness	Cognitive (CRT)	Cognitive (knowledge)
Time now	1								
Time later	0.621***	1							
Present bias	-0.390***	0.480***	1						
Risk aversion	-0.117	-0.049	0.074	1					
Uncertanity aversion	-0.102	-0.110	-0.015	0.600***	1				
Cooperativeness	0.057	0.006	-0.057	-0.175**	-0.117	1			
Competitiveness	0.033	-0.098	-0.151*	-0.101	-0.088	-0.002	1		
Cognitive (CRT)	-0.117	-0.150*	-0.046	0.266***	0.367***	-0.036	0.027	1	
Cognitive (knowledge)	0.040	0.049	0.013	0.051	0.034	0.107	-0.000	0.149*	1
Female	0.03	0.144*	0.133	-0.175**	-0.289***	0.120	-0.112	-0.386***	-0.098
Observations	242								

* p<0.05,

** p<0.01,

*** p<0.001

Similarly, to Dean and Ortoleva [76], we find significant positive pairwise correlations (about 0.6) between present and future time preferences and between risk and uncertainty aversion, but while they report significant positive relationship between measures of risk/uncertainty attitude and measured discount rates (Time now and Time later), we do not find such associations. Another result that we share with Dean and Ortoleva [76] is the significant negative relationship between measured discount rate and cognitive abilities (that they call intelligence, for details see S2 Appendix). Frederick [77] reports that those who obtain a higher score in the CRT, are generally more patient. We see this finding in our measures as Time later is negatively related to cognitive abilities. Frederick [77] also reports that in gambles involving gains (as our decisions 2 and 3) those with higher CRT score were more willing to gamble, a finding that neither we nor Dean and Ortoleva [76] share. This finding has been confirmed by Dohmen et al. [3]. However, Andersson et al. [85] show that this relationship may be spurious. In our experimental tasks, participants with higher CRT score were betting lower amounts in decision 2 and 3. In our data, cognitive abilities correlate positively with risk and uncertainty aversion, and with time preferences at the longer horizon. Dean and Ortoleva [76] only find negative correlation between cognitive abilities and time preferences at the more distant time horizon, but no significant correlation between cognitive abilities and risk or uncertainty. We note that there are studies that find a significant association between cognitive skills and uncertainty attitudes [86] and cognitive skills and cooperation [87,88]. In line with the literature, we also observe that female are more risk/uncertainty averse [34] and perform worse in the CRT [77]. We also find some weak associations for which we are not aware of any findings in the literature and we do not have a good explanation either. Thus, competitive people tend to be less present biased, and that cooperativeness goes negatively with risk aversion.

Overall, the raw measures of the preferences that we study are in line with those found in the literature, and the correlations between our measures resemble in most instances those in Dean and Ortoleva [76].

## Results

### Bivariate associations

We begin our analysis studying linear correlations and non-linear bivariate associations between cognitive and non-cognitive skills and the academic outcomes. Then, we proceed with regressions that allow both for linear and non-linear relationships.

#### Linear relationships

Our main question is whether and how the preferences that we measured are associated with school performance. Remember that we measure school performance using the exam results and GPA in the semester when the exam was taken.

**Fig 1:**
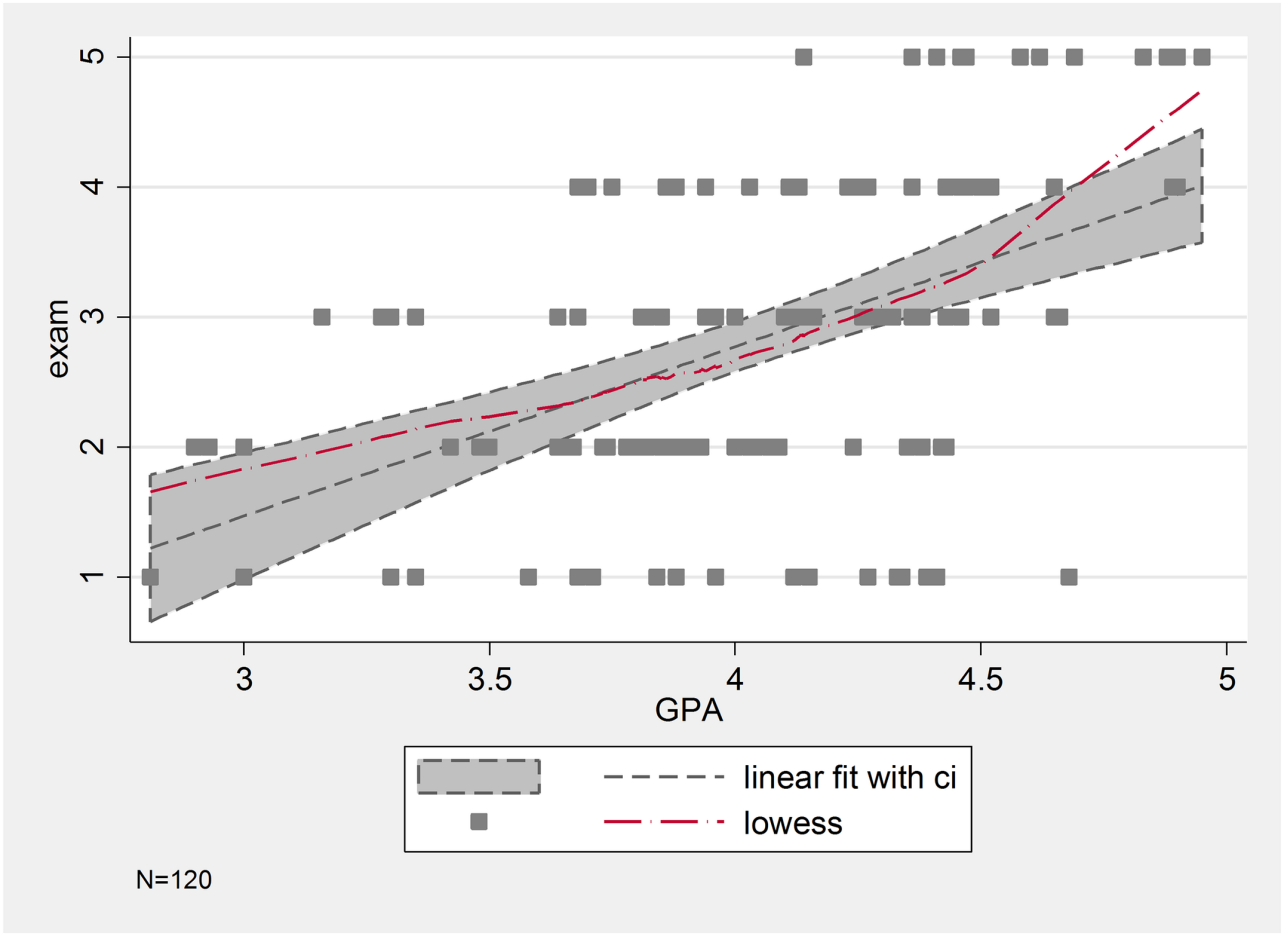
Linear and nonparametric association between the outcome variables.

We note that pairwise correlations indicate that our performance measures are highly positively correlated (p-value<0.001) (see Fig 1).

In Table 4, we show a correlation table indicating the association between our performance measures and the preferences and traits that we measured in our experimental tasks.

**Table 4:** Correlation between preferences and academic performance.

	Time now	Time later	Present bias	Risk aversion	Uncertainty aversion	Cooperativeness
Exam	-0.083	-0.135	-0.061	0.141	0.173*	0.094
GPA	-0.024	-0.089	-0.075	0.112	0.005	0.005
	Competitiveness	Cognitive (CRT)	Cognitive (knowledge)	Female	Exam	
Exam	0.001	0.310***	0.101	-0.003	1	
GPA	0.105	0.178*	0.122	0.208*	0.484***	

*p<0.05,

**p<0.01,

***p<0.001

Most of our measured preferences do not seem to correlate well with school performance. Only uncertainty aversion has a marginally significant linear relationship with exam grades. However, unsurprisingly, individuals with better cognitive abilities perform better. Burks et al. [44] also report that cognitive skill measures predict academic achievement of university students. Note, however, that only the CRT measure correlates well with academic outcomes, our other—self-made—measure of cognition is a much weaker tool. Notice also that the Cognitive Reflection Test mainly involves mathematical content, so it is suitable to detect cognitive capacity related to mathematical abilities. The exam (remember the subject was Economics) contained some calculations (hence requiring mathematical skills), but also conceptual questions, so it was balanced in this sense. It is also interesting that while CRT correlates both with the exam and the GPA, the former correlation is clearly stronger. In the Introduction we hinted at the possibility that a multiple-choice test with clear good answers can be seen as an objective measure. Cognitive abilities might be more important by objective measures than by the GPA where subjectivity may play a larger role (therefore increasing the importance of non-cognitive skills). The fact that cognitive skills (measured by the CRT) associate clearly more with the exam results than with the GPA is a suggestive signal that the role of cognitive abilities is related to the objectivity of the academic outcome.

Regarding our hypotheses described in section 2, the linear relationships seen above do not reveal a consistent and significant effect of the studied non-cognitive skills on the academic outcome. Therefore, next, we investigate if there are non-linear associations.

#### Non-linear relationships

To allow for possible non-linearities, we consider local linear regressions that can be represented in an illustrative way using lowess smoothing. Below, we show all the lowess curves that depict the relationship between the preferences and the performance measures, along with their linear associations. Note that on the x-axis in all figures we report minimum, median and maximum values in the given bivariate relation. These curves confirm mainly the findings seen in correlations, but also give way to other, non-linear associations, which might be very important for future research.

**Fig 2:**
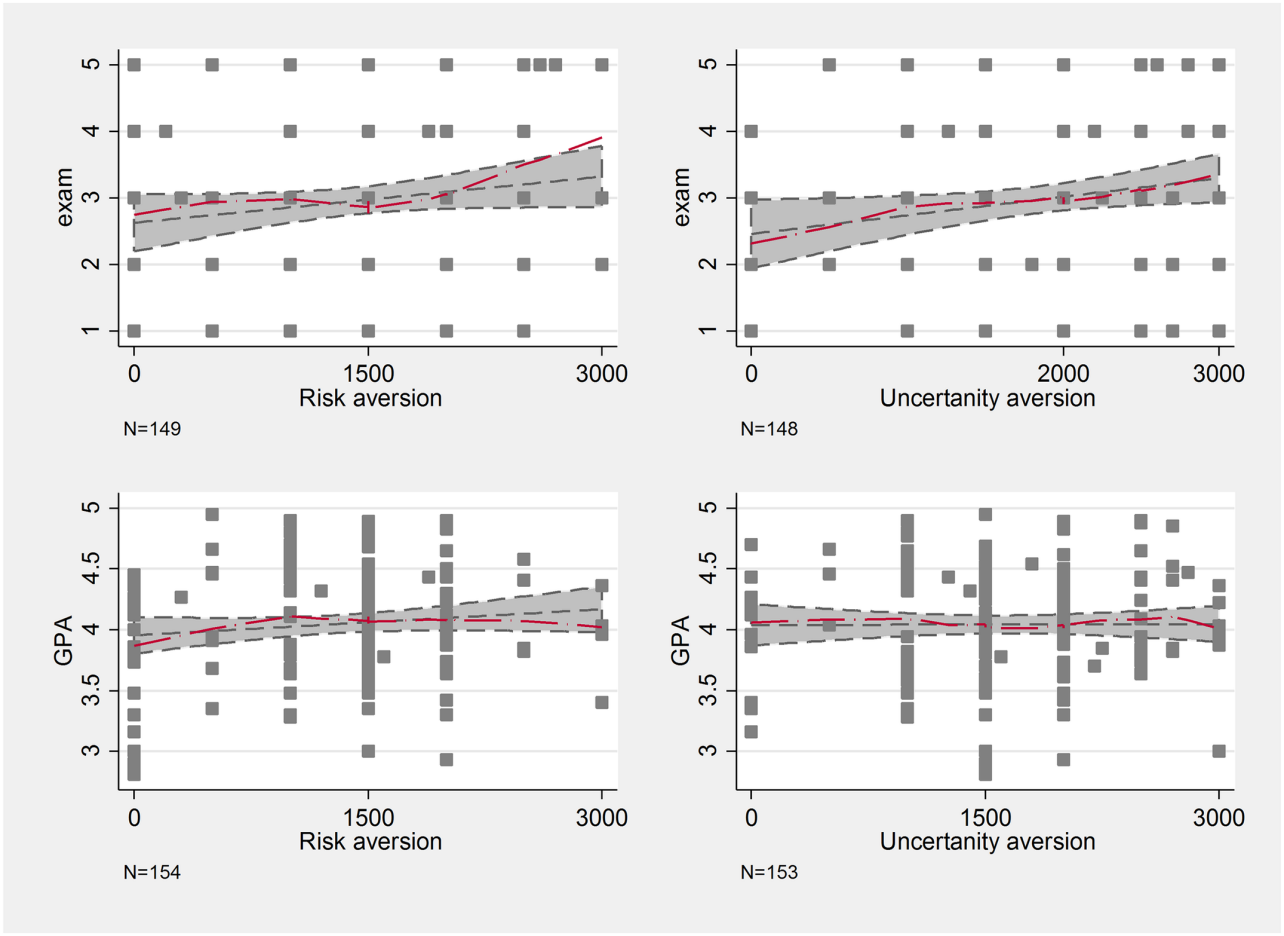
Lowess curves of risk and uncertainty aversion and school performance.

Risk and uncertainty aversion seem to have a positive effect on exam results, especially on the upper end of the distribution, but when considering GPA, the influence appears to be much smaller (Fig 2). These associations are intuitive. At Eötvös Loránd University (our University) each student has a chance to retake all exams once per semester (and one exam per semester twice) without retaking the whole course, and there is no penalization for failing a previous exam. As our Economics exam is a twenty-item four-choice multiple choice test, more risk-tolerant students might go for their first exam less prepared hoping that they can get a pass without much effort. Naturally, this can negatively affect their received exam grades. Many of their other exams follow different grading techniques: home assignments, oral exams or continuous testing, which makes risk-taking less beneficial; hence the much weaker effect of risk preferences on GPA. (But note that due to this logic and to the fact the Economics is one grade in the GPA we would still expect a positive association between risk aversion and GPA).

**Fig 3:**
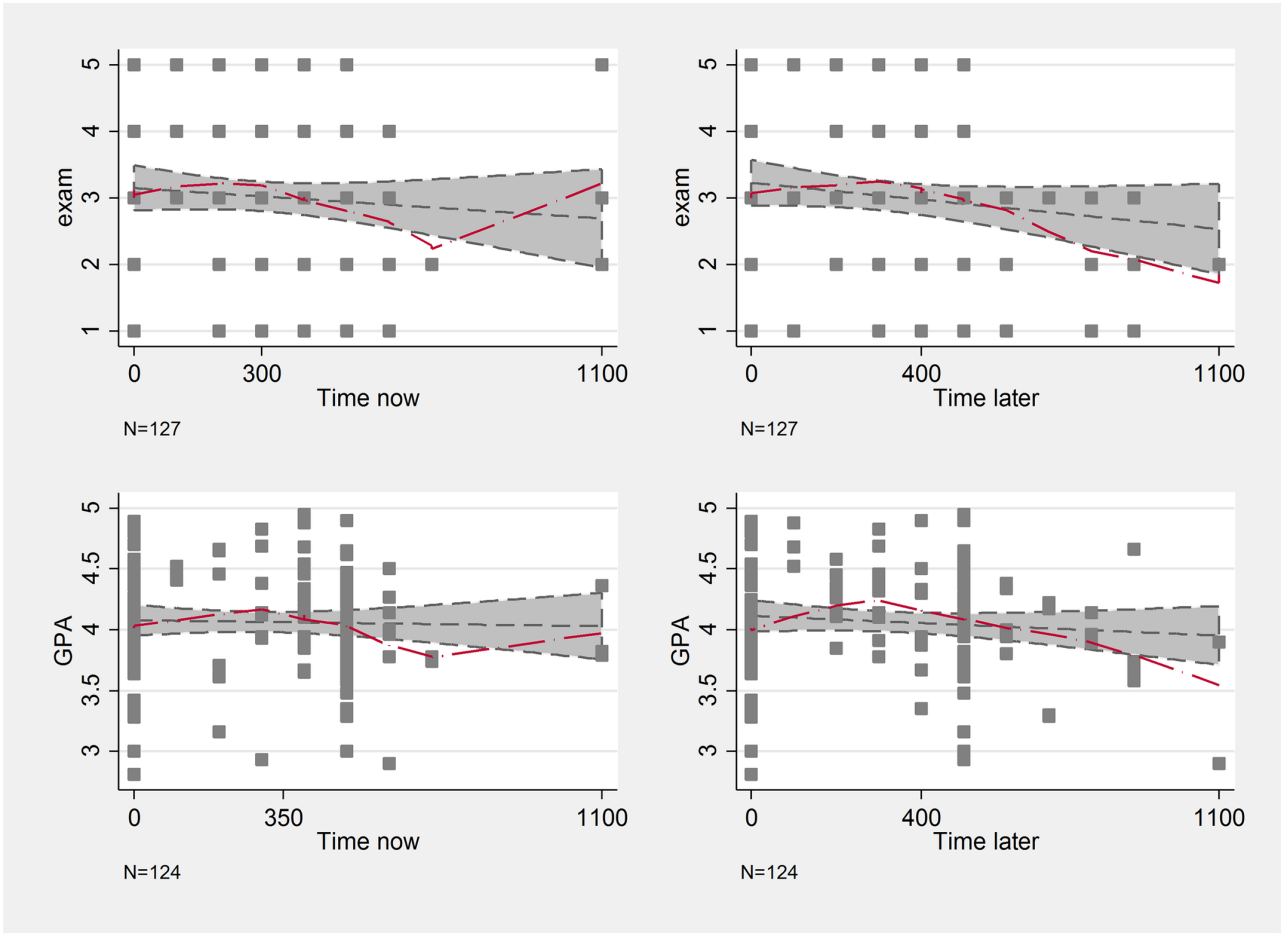
Lowess curves of time preferences and school performance.

**Fig 4:**
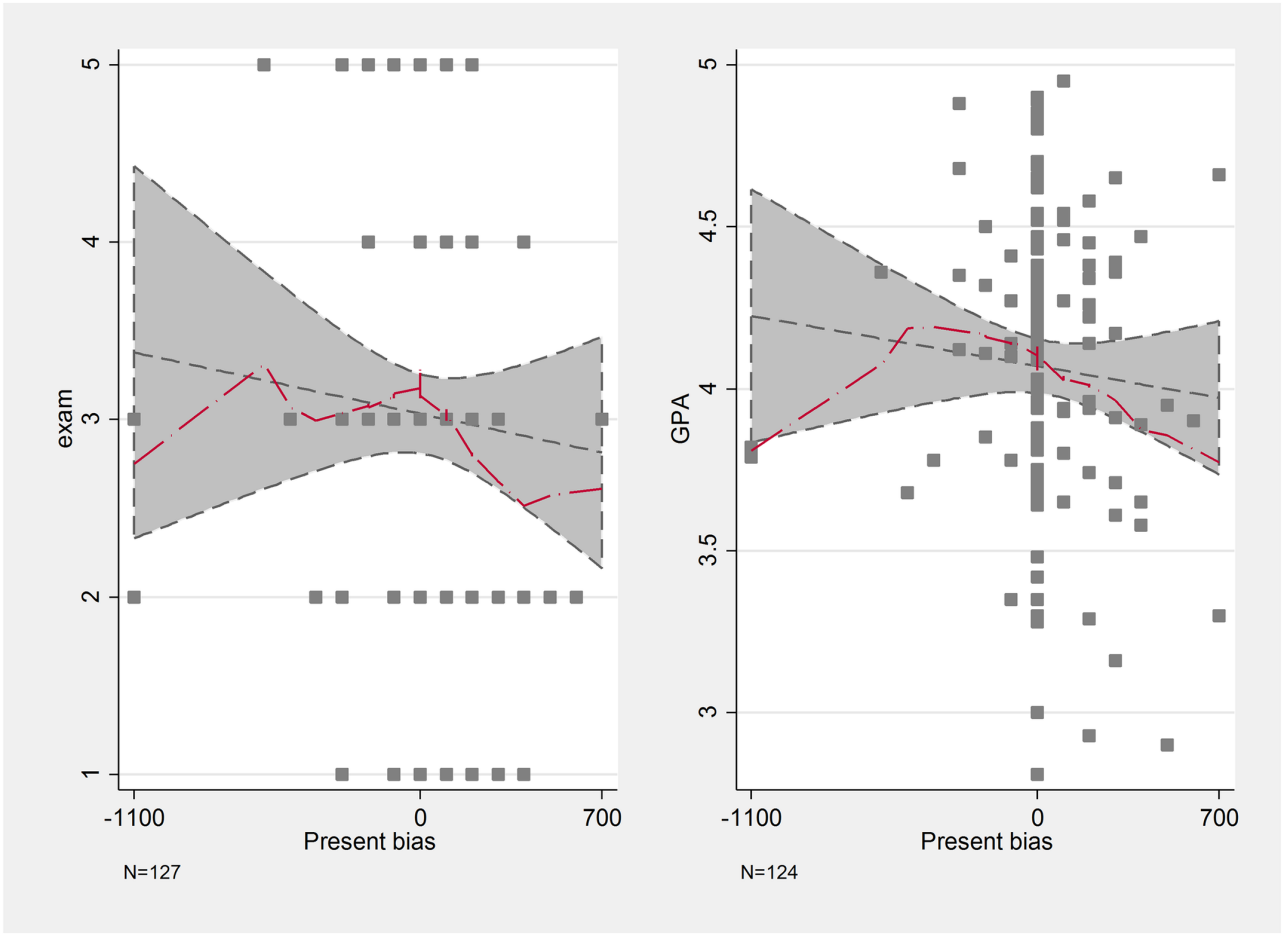
Lowess curves of present bias and school performance.

The measures related to time preferences behave as we expected based on the literature. More precisely, patience is negatively related to exam results, but only on the upper half of the distribution. Students, whose weekly alternative costs are between 0 and 300 forints (that is, those who switch from the later 3500 Ft to the earlier payment when those payments are at least 3200 Ft), perform similarly well, but those, who accept less instead of the 3500 HUF later payment, on average perform worse both on the exam and have worse GPA. Some outliers blur this relationship in the Time now case. This suggests that more impatient students tend to have worse grades (Fig 3), a finding that is in line with most results in the literature. This non-linear relationship is especially striking between present bias and educational outcomes. In Fig 4, we represent on the x-axis the difference between the switching points on the two horizons. Those participants who are in the negative range are future-biased as they are relatively more patient now. If this difference is zero, then the participant is time-consistent, while in the positive range we have the present-biased individuals. The more present-biased a student is, the worse his/her performance is. The performance of future-biased students does not differ from their time-consistent peers.

**Fig 5:**
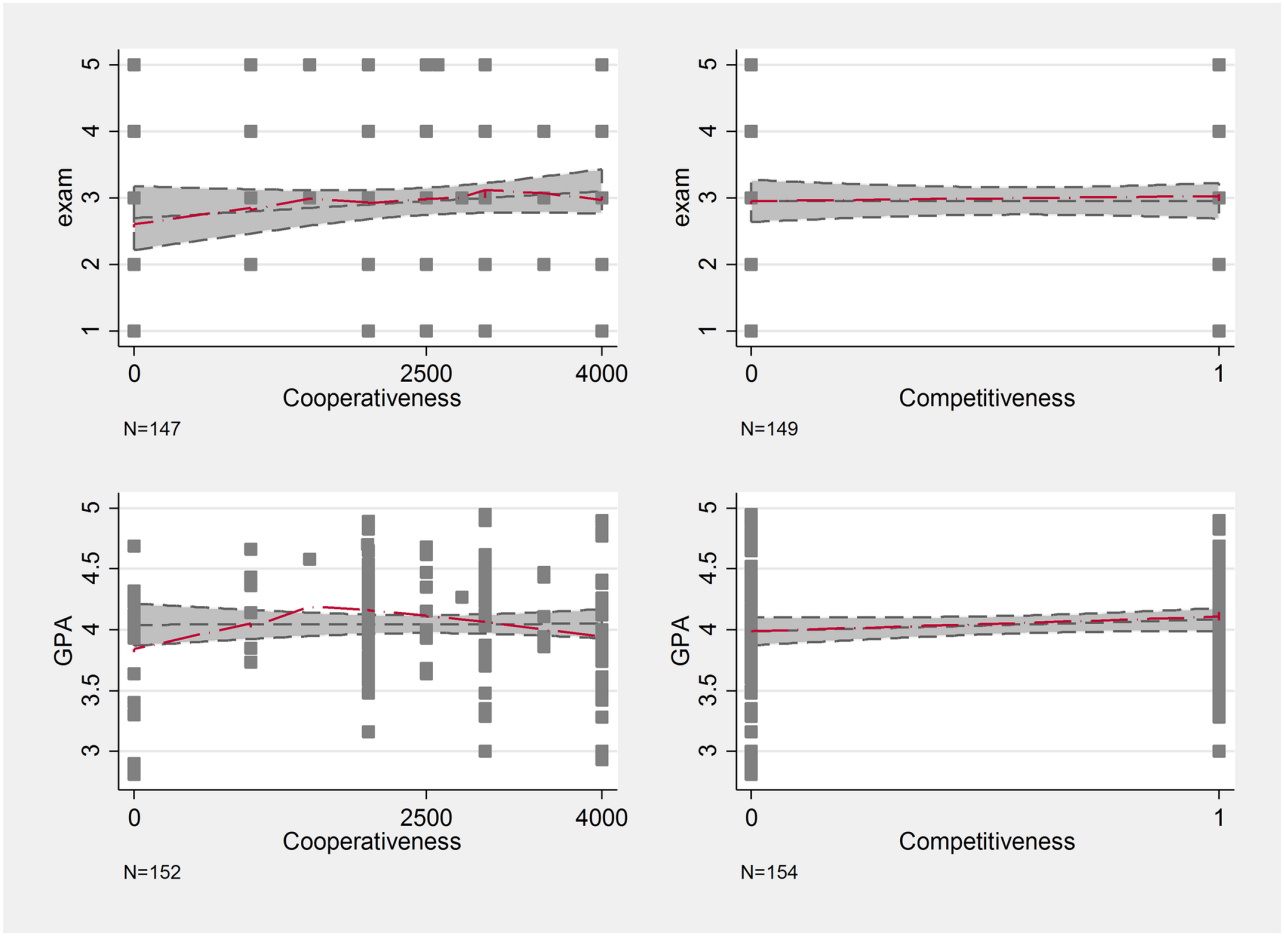
Lowess curves of cooperativeness, competitiveness and school performance.

We have assumed that cooperativeness has a positive effect on grades. Looking at the exam results, we see this positive linear slope, but the difference between the not-at-all cooperative students and the very cooperative ones are slim (see Fig 5). However, cooperativeness seems to have a non-linear relationship with GPA. Students that offer little less than the half of their total endowment (2000 HUF) for the others have the highest GPA, and anyone under or over this amount perform worse (note that the median is around 2500). While it is intuitive that cooperativeness should have a higher effect on the GPA than on a single exam grade, the non-linear effect of cooperativeness is non-trivial and not in line with our Hypothesis 3.

Competitiveness looks to have no effect when considering the exam results, but there is a slight positive relationship regarding GPA, hinting at the possibility that more competitive students have better GPA. This is in line with our Hypothesis 4. Note, that the quiz used to measure competitiveness included the small cognitive test; thus, it might be wise to control for cognitive test scores to see a less biased relationship between competitiveness and grades (see below).

**Fig 6:**
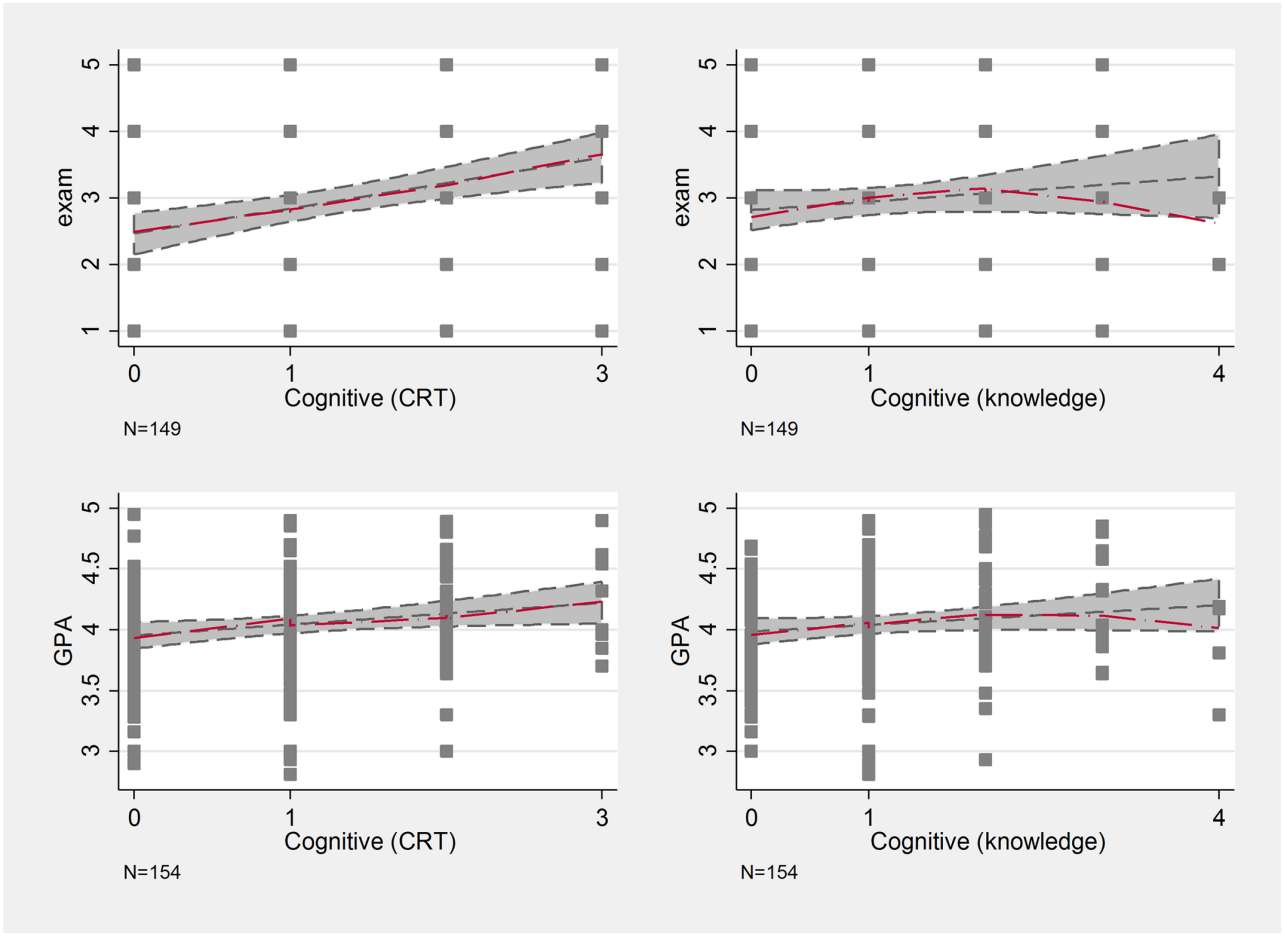
Lowess curves of cognitive tests and school performance.

Cognitive abilities, have an especially strong positive relationship with grades. Cognitive abilities measured by the CRT have a clear positive effect (in line with expectations), but the other questions that made up the quiz do not seem to be clearly related to academic performance (see Fig 6).

The previous analysis indicates that some of the preferences that we study have a non-linear association with the academic outcome. Risk attitudes associate only with exam results (in the expected way) of those who most tolerate risk. More impatient students tend to have worse school performance, but the negative effect kicks in only when students are sufficiently impatient. We find a strange non-linear relationship between our social preference measure and educational outcome that is not in line with our hypothesis. The effect of competitiveness on school performance is as expected, but rather small. Note that even if we allow for non-linearities, the analysis may not reveal relationships if we do not control for other variables. Hence, we proceed with the regression analysis.

### Regressions

#### Linear relationships

As a next step, we see if the previous findings hold when we control for other variables, so we run OLS regressions. The lowess curves indicate that the relationships are linear in some cases, but non-linear in others. Whenever the bi-variate relations are linear, we will use the linear approximation, but for some preferences we will experiment with non-linear functional forms as well.

For regressions on the exam result we include exam-time fixed effects, and also cluster the standard errors on exam time. Although the Economics exams are multiple choice tests, they are not standardized, so it might be that the exam is harder at one time than at another. Also students from different faculties—Social Science and Computer Science—take exams at different times. By including exam time fixed effects we intend to control for these two potential confounding effects.

For regressions on the GPA we included fixed effects for the study major. Assuming different departments grade differently and also that different majors have different subjects (both affecting the GPA), this might—and sometimes do—change the results. We have also utilized the number of credits that students have gained: using the number of credits as frequency weights we give a larger weight to a student that takes more classes than another with fewer credits. On average students gained little less than 30 credits in our sample (mean = 29.9, median = 30, 1pct = 12, 99pct = 38, sd. = 4.17), of this the Economics exam was worth 3 credits. We have clustered the standard errors on student level within these credit-weighted regressions. As a robustness check we also report the unweighted regressions.

In all regressions below, we control for the gender of the student. This is especially important as gender is not balanced across faculties and majors.

As can be seen in Tables 5 to 7 below, the regressions confirm most of the previous findings. The most solid result is that cognitive abilities affect positively and significantly both exam results and the GPA (both when weighted and when not). Competitiveness seems to associate positively and significantly with GPA (both weighted and unweighted) once the gender and study-major fixed-effects are controlled for, and the point estimates of the exam grades are very similar to the GPA point estimates. Uncertainty / risk aversion is positively and significantly related to better exam results / GPA in the unweighted / weighted regression. These linear regressions fail to detect any association between patience, present bias, cooperativeness and the educational performance.

**Table 5:** Linear regressions of exam grades and preferences—Without weights.

Outcome: Exam	(1)	(2)	(3)	(4)	(5)	(6)	(7)	(8)	(9)
Dependent variable:	Time now (100HUF)	Time later (100HUF)	Risk aversion (100 HUF)	Uncertanity aversion (100 HUF)	Present bias	Cooperativeness (100 HUF)	Competitiveness	Cognitive (knowledge)	Cognitive (CRT)
Main coefficient (see column)	-0.036	-0.071	0.023	0.029*	-0.045	0.008	0.114	0.146*	0.487***
	(0.056)	(0.04)	(0.015)	(0.015)	(0.061)	(0.01)	(0.353)	(0.078)	(0.087)
Female	-0.034	0.026	-0.027	0.074	-0.0233	-0.057	-0.006	0.005	0.326
	(0.426)	(0.413)	(0.340)	(0.321)	(0.453)	(0.386)	(0.385)	(0.367)	(0.271)
Constant	3.160***	3.236***	3.363***	3.280***	3.062***	2.782***	2.910***	2.810***	2.128***
	(0.429)	(0.373)	(0.375)	(0.356)	(0.307)	(0.382)	(0.391)	(0.262)	(0.209)
Observations	121	121	139	138	121	138	139	139	139
R-squared	0.093	0.108	0.133	0.140	0.095	0.124	0.117	0.127	0.233
exam time FE	y	y	y	y	y	y	y	y	y
Weights	n	n	n	n	n	n	n	n	n

Robust standard errors clustered on exam x time level are in parentheses.

*** p<0.01,

**p<0.05,

* p<0.1

**Table 6:** Linear regressions of GPA and preferences—Without weights.

Outcome: GPA	(1)	(2)	(3)	(4)	(5)	(6)	(7)	(8)	(9)
Dependent variable:	Time now (100HUF)	Time later (100HUF)	Risk aversion (100 HUF)	Uncertanity aversion (100 HUF)	Present bias	Cooperativeness (100 HUF)	Competitiveness	Cognitive (knowledge)	Cognitive (CRT)
Main coefficient (see column)	-0.0003	-0.015	0.011	0.001	-0.018	0.0004	0.150*	0.095***	0.039
	(0.032)	(0.026)	(0.006)	(0.002)	(0.014)	(0.002)	(0.061)	(0.016)	(0.028)
Female	0.150	0.163*	0.195***	0.210**	0.166*	0.204**	0.239***	0.234**	0.212**
	(0.076)	(0.063)	(0.041)	(0.049)	(0.071)	(0.052)	(0.043)	(0.054)	(0.049)
Constant	3.956***	3.992***	4.108***	3.919***	3.949***	3.894***	3.791***	3.784***	3.855***
	(0.133)	(0.114)	(0.104)	(0.033)	(0.056)	(0.064)	(0.045)	(0.044)	(0.027)
Observations	118	118	142	141	118	141	142	142	142
R-squared	0.038	0.045	0.084	0.054	0.046	0.052	0.080	0.088	0.062
major FE	y	Y	y	y	y	y	y	y	y
freq. weights	n	N	n	n	n	n	n	n	n

Robust standard errors clustered on faculty level are in parentheses.

*** p<0.01,

** p<0.05,

* p<0.1

**Table 7:** Linear regressions of GPA and preferences—With weights.

Outcome: GPA	(1)	(2)	(3)	(4)	(5)	(6)	(7)	(8)	(9)
Dependent variable:	Time now (100HUF)	Time later (100HUF)	Risk aversion (100 HUF)	Uncertanity aversion (100 HUF)	Present bias	Cooperativeness (100 HUF)	Competitiveness	Cognitive (knowledge)	Cognitive (CRT)
Main coefficient (see column)	-0.004	-0.014	0.012**	0.001	-0.012	0.0004	0.139*	0.099**	0.048
	(0.016)	(0.019)	(0.005)	(0.005)	(0.019)	(0.004)	(0.082)	(0.04)	(0.037)
Female	0.154	0.167	0.194**	0.208**	0.166	0.203**	0.235**	0.231**	0.212**
	(0.108)	(0.106)	(0.09)	(0.095)	(0.110)	(0.097)	(0.095)	(0.097)	(0.095)
Constant	3.993***	4.016***	4.143***	3.941***	3.975***	3.921***	3.827***	3.810***	3.871***
	(0.121)	(0.116)	(0.118)	(0.107)	(0.097)	(0.138)	(0.108)	(0.104)	(0.096)
Observations	118	118	142	141	118	141	142	142	142
Observations weighted with credits	3,572	3,572	4,274	4,245	3,572	4,246	4,274	4,274	4,274
R-squared	0.039	0.045	0.083	0.052	0.043	0.051	0.074	0.089	0.065
major FE	y	y	y	y	y	y	y	y	y
freq. weights	y	y	y	y	y	y	y	y	y

Robust standard errors clustered on the individual level are in parentheses.

*** p<0.01,

** p<0.05,

* p<0.1

#### Non-linear relationships

As we have argued above, time preferences seem to have a non-linear relationship with grades. Both patience (Time now and Time later) as well as their difference, the present bias, seem to correlate strongly with exam results and GPA but only on specific intervals on the lowess curves. In the regressions below we show that this partial relationship is also statistically significant. As Fig 2c has suggested, present biased students might behave differently those who do not exhibit present bias. In Table 8 below we estimate the effect of present / future bias on the outcomes. For the Time now/Time later variables we have used a squared term to identify potential diminishing returns to scale, as hinted by Fig 2b (see Table 9).

**Table 8:** Regressions with present bias.

VARIABLES	(1)	(2)	(3)
	exam	GPA	GPA
Future bias (100HUF)	0.028	0.008	0.011
	(0.063)	(0.024)	(0.017)
Present bias (100HUF)	-0.131**	-0.047	-0.044
	(0.042)	(0.023)	(0.034)
Female	-0.005	0.167*	0.179
	(0.444)	(0.071)	(0.108)
Constant	3.162***	3.989***	3.990***
	(0.312)	(0.08)	(0.101)
Observations	121	118	118
Observations weighted with credits			3,423
R-squared	0.110	0.061	0.060
exam time FE	y		
Weights	n	n	y
faculty FE		y	y
errors clustered on	exam x time level	faculty level	individual level

Robust standard errors in parentheses.

*** p<0.01,

** p<0.05,

* p<0.1

**Table 9:** Regressions with Time now.

VARIABLES	(1)	(2)	(3)	(4)	(5)	(6)
	exam	exam	GPA	GPA	GPA	GPA
Time now (100HUF)	-0.036	-0.052	-0.0003	0.034	-0.0005	0.036
	(0.056)	(0.113)	(0.032)	(0.058)	(0.016)	(0.034)
Time now, squared (100 HUF)		0.002		-0.005		-0.005
		(0.013)		(0.004)		(0.004)
Female	-0.034	-0.031	0.150	0.145	0.165	0.158
	(0.426)	(0.420)	(0.076)	(0.078)	(0.104)	(0.102)
Constant	3.160***	3.171***	3.956***	3.931***	3.956***	3.931***
	(0.429)	(0.463)	(0.133)	(0.154)	(0.116)	(0.122)
Observations	121	121	118	118	118	118
Observations weighted with credits					3,423	3,423
R-squared	0.093	0.093	0.038	0.047	0.039	0.049
exam time FE	y	y				
Weights	n	n	n	n	y	y
major FE			y	y	y	y
errors clustered on	exam time level		faculty level		individual level	

Robust standard errors in parentheses.

*** p<0.01,

** p<0.05,

* p<0.1

Table 8 below shows that present bias correlates significantly with exam results and that the correlation with GPA is also negative but insignificant on conventional levels. Moreover, Table 10 also shows that the squared function of Time later significantly relates to both exam grades and the GPA (and that the estimation with Time now has similar but insignificant point estimates due to a couple of outliers at the upper end).

**Table 10:** Regressions with Time later.

VARIABLES	(1)	(2)	(3)	(4)	(5)	(6)
	exam	exam	GPA	GPA	GPA	GPA
Time later (100HUF)	-0.0709	0.0734	-0.0145	0.0898	-0.0125	0.0942**
	(0.0403)	(0.0910)	(0.0263)	(0.0622)	(0.0180)	(0.0390)
Time later, squared (100 HUF)		-0.0194*		-0.0136**		-0.0140***
		(0.00894)		(0.00443)		(0.00480)
Female	0.0260	0.0215	0.163*	0.177*	0.177*	0.189*
	(0.413)	(0.407)	(0.0631)	(0.0679)	(0.102)	(0.0983)
Constant	3.236***	3.116***	3.992***	3.892***	3.987***	3.885***
	(0.373)	(0.395)	(0.114)	(0.165)	(0.111)	(0.113)
Observations	121	121	118	118	118	118
Observations weighted with credits					3,423	3,423
R-squared	0.108	0.126	0.045	0.120	0.044	0.121
exam time FE	y	y				
Weights	n	n	n	n	y	y
major FE			y	y	y	y
errors clustered on	exam time level		faculty level		individual level	

Robust standard errors in parentheses.

*** p<0.01,

** p<0.05,

* p<0.1

Besides the face value of the lowess curves above these relations are also theoretically sound: we find that large present bias has a negative association with exam grades (but not GPA), but also that future bias has no relation to either exam grades or GPA. Also, patience (that is, our Time later variable) matters especially for those, who are very impatient. Our estimates of time preference—as any—is inherently imperfect; thus we do not find much difference with not or little impatient student. However, students who value the future much less than the present also perform worse in school, as school is a very typical investment in the future.

The other preference, where we have seen a possible non-linear effect was cooperativeness. As seen in the regressions in Table 11 below a quadratic function describes the data better than the linear one, especially with GPA as the dependent variable. Based on these estimates those, who have contributed little over 2000HUF (half of the total endowment) to the public good perform around 0.3 grades better than those, who have contributed nothing or everything to the pot. This is a significant difference. While based on the literature we have a good story for the non-linearities seen in the case of time preference, we lack any good explanation why an intermediate amount of contribution in the public goods game is associated with better GPA.

**Table 11:** Regressions with cooperativeness.

VARIABLES	(1)	(2)	(3)	(4)	(5)	(6)
	Exam	exam	GPA	GPA	GPA	GPA
Cooperativeness (100 HUF)	0.008	0.027	0.0004	0.028***	0.0004	0.028**
	(0.01)	(0.033)	(0.002)	(0.004)	(0.004)	(0.011)
Cooperativeness squared (100 HUF)		-0.0004		-0.0006***		-0.0006***
		(0.0007)		(0.0001)		(0.0002)
Female	-0.057	-0.066	0.204**	0.187**	0.215**	0.197**
	(0.386)	(0.379)	(0.052)	(0.06)	(0.094)	(0.087)
Constant	2.782***	2.655***	3.894***	3.718***	3.892***	3.717***
	(0.382)	(0.498)	(0.064)	(0.068)	(0.141)	(0.154)
Observations	138	138	141	141	141	141
Observations weighted with credits					4,090	4,090
R-squared	0.124	0.127	0.052	0.107	0.054	0.109
exam time FE	Y	y				
weights	N	n	n	n	y	y
major FE			y	y	y	y
errors clustered on	exam time level		faculty level		individual level	

Robust standard errors in parentheses.

*** p<0.01,

** p<0.05,

* p<0.1

#### Multivariate linear regressions

Although the number of observations in our study is not very large, and thus running multivariate regressions risks the problem of overidentification, we still experimented with the inclusion of more than one preference in the regressions.

First of all, as we have shown above both risk aversion and non-linear time preferences (or present bias) associate with grades. These two preferences might signal the same non-cognitive trait, as impatience or present bias can easily stem from an inherent dispreference towards risk (cf. future is risky). Including both preferences in one regression (see Table 12), however, does not affect the point estimates or their significance. Note that we use Time later to proxy impatience as it suffers less from outliers. This suggests that risk aversion and time preferences are indeed different non-cognitive traits [28], which both relate, independently, to school performance.

**Table 12:** Multivariate regressions with time and risk preferences.

VARIABLES	(1)	(2)	(3)	(4)	(5)	(6)
	exam	GPA	GPA	exam	GPA	GPA
Future bias (100HUF)	0.011	0.006	0.007			
	(0.067)	(0.027)	(0.018)			
Present bias (100HUF)	-0.136**	-0.049	-0.045			
	(0.043)	(0.025)	(0.035)			
Time later (100HUF)				0.073	0.083	0.086**
				(0.099)	(0.059)	(0.037)
Time later, squared (100 HUF)				-0.019*	-0.013**	-0.013***
				(0.01)	(0.004)	(0.004)
Risk aversion (100 HUF)	0.028*	0.012	0.014**	0.027	0.011	0.012**
	(0.015)	(0.007)	(0.006)	(0.017)	(0.006)	(0.005)
Female	0.008	0.168**	0.180*	0.028	0.176**	0.189**
	(0.397)	(0.050)	(0.1)	(0.369)	(0.049)	(0.092)
Constant	3.589***	4.200***	4.226***	3.526***	4.087***	4.108***
	(0.414)	(0.078)	(0.130)	(0.478)	(0.087)	(0.136)
Observations	121	118	118	121	118	118
			3,423			3,423
R-squared	0.136	0.097	0.104	0.150	0.147	0.156
exam time FE	y					
weights	n	n	y	n	n	y
major FE		y	y	y	y	y
errors clustered on	exam time level		faculty level		individual level	

Robust standard errors in parentheses.

*** p<0.01,

** p<0.05,

* p<0.1

On a different note, one might argue that competitiveness matters only through higher cognitive test scores. Remember, competitiveness was measured using a small cognitive test. If a student knew that s/he was performing badly on that small test, s/he was less likely to opt for the competitive payment. Thus, including both cognitive scores in the regression could show whether they alter the association of competitiveness and performance (see Table 13). Apparently, this association does not depend on the cognitive scores of the students. Students, who opted for the competitive payment on average receive 0.13 higher grades, even after controlling for their cognitive test scores. Moreover, this effect is marginally significant for the GPA, and similar in size in all specifications.

**Table 13:** Multivariate regressions with competitiveness and cognitive abilities.

VARIABLES	(1)	(2)	(3)
	exam	GPA	GPA
Competitiveness	0.136	0.143*	0.132
	(0.275)	(0.065)	(0.081)
Cognitive (knowledge)	0.096	0.036	0.042
	(0.087)	(0.031)	(0.035)
Cognitive (CRT)	0.475***	0.082***	0.086**
	(0.089)	(0.017)	(0.041)
Female	0.350	0.264***	0.275***
	(0.281)	(0.047)	(0.096)
Constant	1.947***	3.648***	3.642***
	(0.318)	(0.056)	(0.135)
Observations	139	142	142
			4,120
R-squared	0.240	0.115	0.118
exam time FE	y		
weights	n	n	y
major FE		y	y
errors clustered on	exam time level	faculty level	individual level

Robust standard errors in parentheses.

*** p<0.01,

** p<0.05,

* p<0.1

Finally, plugging (almost) all variables in the regression (Table 14) below does not really change the conclusions. Of course, as the power of our analysis is rather small, it eliminates some significant effects, and while some point estimates also decreased a little (e.g., time preferences), the main direction of associations remain, suggesting we were quite successful in finding and measuring independent preferences. Previous studies focus on fewer preferences that we do (e.g., Burks et al. [44] consider time and risk preferences, while Non and Tempelaar [42] study only time preferences) and through correlations between preferences omitted from those studies, one might argue that there is bias in their estimates. Our results suggest that these preferences are in fact rather independent, lending more credence to their findings.

**Table 14:** Multivariate regressions with (almost) all preferences.

VARIABLES	exam	GPA	GPA	exam	GPA	GPA
Future bias (100HUF)	0.023	0.015	0.016			
	(0.049)	(0.018)	(0.015)			
Present bias 0 (100HUF)	-0.130***	-0.029	-0.027			
	(0.04)	(0.033)	(0.032)			
Time later (100HUF)				-0.053	0.057	0.06
				(0.100)	(0.04)	(0.037)
Time later, squared (100 HUF)				-0.004	-0.009**	-0.009*
				(0.013)	(0.003)	(0.005)
Risk aversion (100 HUF)	0.017	0.011	0.012**	0.016	0.011	0.011**
	(0.017)	(0.008)	(0.006)	(0.02)	(0.008)	(0.006)
Cooperativeness (100 HUF)	0.05	0.039**	0.038***	0.051	0.033**	0.032***
	(0.046)	(0.013)	(0.011)	(0.053)	(0.012)	(0.011)
Cooperativeness squared (100 HUF)	-0.001	-0.001**	-0.001***	-0.001	-0.0001**	-0.001***
	(0.001)	(0.0003)	(0.0002)	(0.001)	(0.0002)	(0.0003)
Competitiveness	0.206	0.191***	0.178*	0.229	0.181***	0.163*
	(0.376)	(0.035)	(0.094)	(0.385)	(0.033)	(0.093)
Cognitive (CRT)	0.456***	0.077*	0.082*	0.468***	0.076*	0.079*
	(0.092)	(0.034)	(0.049)	(0.104)	(0.03)	(0.047)
Cognitive (knowledge)	0.135	-0.004	0.001	0.124	-0.008	-0.003
	(0.107)	(0.048)	(0.037)	(0.086)	(0.05)	(0.038)
Female	0.275	0.190**	0.201**	0.329	0.202***	0.213**
	(0.391)	(0.045)	(0.094)	(0.397)	(0.04)	(0.089)
Constant	1.912**	3.695***	3.712***	1.924**	3.668***	3.688***
	(0.640)	(0.04)	(0.184)	(0.704)	(0.072)	(0.180)
Observations	120	117	117	120	117	117
			3,393			3,393
R-squared	0.285	0.231	0.231	0.293	0.251	0.253
exam time FE	y			y		
Weights	n	n	y	n	n	y
major FE		y	y		y	y
errors clustered on	exam time level	faculty level	individual level	exam time level	faculty level	individual level

Robust standard errors in parentheses.

*** p<0.01,

** p<0.05,

* p<0.1

## Discussion and conclusion

Our paper has aimed to be among the frontrunners in connecting several experimental measures of preferences with real-life measures of school success. Being an exploratory pilot study, we experimented with four different preferences, which, according to the literature and our view, might have an impact on school performance. We measured in an incentivized way risk, time, social and competitive preferences and cognitive abilities of university students and attempted to find associations between these measures and two important academic outcome measures: exam results and GPA.

Based on the previous literature we have set up four testable hypotheses on the association of preferences and grades. We expected (1) patience to have a positive and present bias a negative effect; (2) no effect of risk; (3) positive effect of cooperativeness and (4) positive effect of competitiveness on school performance. Furthermore, we conjectured that cognitive abilities would correlate well with academic performance.

We find consistently that cognitive abilities are very well correlated with school performance. We used the Cognitive Reflection Test [77] to proxy cognitive skills and found that it explains grades on the Economics exam extremely well, and associates well with the more general GPA.

Regarding non-cognitive skills, we find suggestive evidence for many of our measured preferences. However, further research is needed to see if our results are representative (see [89])

First of all, the most emphatic non-cognitive skill in the literature is time preference (see also: conscientiousness, or self-control, or discipline) (Hyp 1). We used two alternative measures of time preference: patience and present bias. We measured patience as a choice between different amounts of money at different points in time. The more individual values future payoffs, the more patient she is. Moreover, if an individual is more impatient on the short-term horizon than on the long-term, then she exhibits present bias that may make it hard for the individual to make efforts immediately, and she tends to procrastinate efforts and costs. While these two indicators of time preference seem to act similarly for both measures of school performance, it is the present bias that explains exam grades relatively better, and patience that explains GPA better. We have also pinpointed that both measures of time preferences have a non-linear relation to school performance. Students exhibiting future bias (those who are more patient on the short run than on the long run) perform similarly to time-consistent students (who are just as patient in the short as in the long run). However, the more present-biased a student is, the worse her grades are. Similarly, patience does not differentiate between very patient and a between little impatient students regarding academic success, however very impatient students seem to perform worse in school.

We have also seen that risk-averse students perform a little better than more risk-tolerant students (Hyp 2). Risk preferences matter a slightly bit more for the exam grade than for the general GPA. In case of multiple choice exams, it makes perfect sense for more risk-tolerant students to go for their first try less prepared, as the possibility of passing as exam just by chance is not zero and because they are not penalized in later exams for failing before. Thus, the expected exam grades of more risk-tolerant tend to be lower, which might affect their GPA as well.

Economists might be pleased to hear that competitiveness seems to matter (Hyp 3). At least this is what we find. Students, who opt for a more competitive payment scheme in our experimental task seem to have a little higher average GPA. This, however, might be confounded by the gender of the students. Unfortunately, we could not study this aspect, as our sample was seriously gender imbalanced across faculties (Social Science students are mainly girls while boys are more likely come from the Computer Science faculty).

Finally, we have also detected that cooperative preferences (Hyp. 4)–the amount of money offered in a public good game—associates strongly with GPA, but in a non-linear way. Students who offered around half of their possible amounts had significantly higher GPAs than those, who offered none or all their money. We do not find this effect for exam grades and have no real theoretical explanations for the non-linear relationship.

An important message of our exploratory study is that the non-cognitive skills often have a non-linear relationship with the measures of academic performance.

All in all, we consider this research a pilot. All our results can be considered preliminary, for three reasons. First, although our sample size is relatively large for an exploratory experimental study, we still run the risk of overidentification when looking at several preferences simultaneously. Second, although our experimental tasks were incentivized, these incentives were relatively small, as we could only pay a couple of students per class based on their decisions. Third, although these preference measures are relatively well established in the behavioural economics literature, they are not at all validated in the economics of education studies. It might be that time preferences—as we measure them—are, but poor proxies of more important non-cognitive traits (as self-discipline, for instance, see [32]), and that cooperativeness should matter not on the individual but on the group level. Thus, we intend this research to generate debate and inspire future research, which could underline or falsify our findings.

Admitting its limits, our study contributes to the literature that shows that both cognitive ability and non-cognitive skills help to predict outcomes in different walks of life. Note that—as mentioned in the Introduction—school performance is a major determinant of success in life, so understanding better all the factors that may affect academic attainment is a relevant endeavour. It is not only the effects of cognitive and non-cognitive skills that matter, but also how they interrelate with family background. There are many studies investigating the heritability of intelligence (and hence cognitive skills) and how it is affected by the socioeconomic status Turkheimer et al. [90,91]. Many studies suggest that low socioeconomic status lowers the heritability of intelligence. Less is known about such interplay between non-cognitive skills and socioeconomic status (notable exceptions include [92,93,94]. Once we know sufficiently well the effects of cognitive and non-cognitive skills on school performance (that to a large extent determines success in life) and how these skills are distributed in the society along the social dimension, can we start to think about interventions [95,96] to improve the skills and augment the likelihood of success in life for future generations.

## Supporting information

S1 Appendix The file S1 Appendix.docx contains the instructions that we used in the experiment. The original instructions were in Hungarian, this supplementary material contains the English translation.
(DOCX)

S2 Appendix The file S2 Appendix.docx contains a detailed description of how Dean and Ortoleva [76] carried out a similar experiment measuring many of the preferences that we also measure. (DOCX)

S1 File The file Supporting Information_Do file.do contains the do file that can be used to replicate our results. (DO)
