# Supplementary material for: Which preferences associate with school performance?—Lessons from an exploratory study with university students
Source: PLoS One. 2018 Feb 16;13(2):e0190163. doi: 10.1371/journal.pone.0190163 (PMC5815576; doi:10.1371/journal.pone.0190163)
Supplement: S1 Appendix — The original instructions were in Hungarian, this supplementary material contains the English translation. (DOCX) [file pone.0190163.s001.docx]

# Appendix A – Instructions

**Dear participant!**

You participate in a scientific research carried out by Dániel Horn and Hubert János Kiss, assistant professors at Eötvös Loránd University.

Participation is VOLUNTARY. You can interrupt the experiment at any moment or refuse to answer the questions without giving any explanation.

Participation is ANONYMOUS. We treat any information gathered in this research confidentially. However, we would like to ask for your NEPTUN code as it is an important part of the research to lint the results of this experiment with your academic results. After linking the data, we will erase your code.

**Neptun code:**  __________

The results of this experiment will be used for research purposes carried out at the Department of Economics, Faculty of Social Sciences, Eötvös Loránd University.

Thanks for participating in this experiment!

# Instructions

In this experiment each participant makes decisions in six different and independent situations. You may earn money depending on your choices! The maximum earning is 7100 Ft.

**At the end of the experiment we select randomly to participants who will receive their earnings in cash.**

**The selection will be as follows:** Each answer sheet has two tags in the upper right corner with a number on them. (Please, check that the numbers on the tag are identical with the number of the answer sheet!) 1) Keep one of the tags; 2) hand in the other tag with the answer sheet after you have completed the experiment. We will select randomly two numbers that identify two participants, and using a die we will select one of the situations in which you made the decisions. The decisions of the chosen participants will determine their earnings. The selection takes place once everybody handed in the answer sheets and earnings will be paid immediately (or depending on the situation in 1,2 or 3 weeks).

Please, do not speak with other participants during the experiment. **Should you have any questions, please turn to the administrators of the experiment!**

# Situation 1

You will be randomly matched with another participant in this room and both of you receive 4000 Ft. You and the other participant independently may contribute any amount (between 0 and 4000 Ft) from this initial endowment to a joint account. After contribution *both* of you will receive the 70% of the total contribution. Your final earning consists of the money *not* contributed to the joint account plus the money received from the joint account.

*For example, if you contribute 3000 Ft, while the other participant 4000 Ft, then there will be 7000 Ft on the joint account. 70% of this amount is 4900 Ft, hence this is the amount that both of you receive from the joint account. Since you contributed 3000 Ft out of 4000 Ft, so you have still 1000 Ft left, and consequently your final earning is 4900+1000=5900 Ft.*

*The next table shows some possible contributions and the ensuing earnings. (Note: you are free to choose any contribution, you are not restricted to the numbers shown in the table.)*

| **Contribution of participant 1** | **Contribution of participant 2** | **Joint account** | **Earning of participant 1** | **Earning of participant 2** |
| --- | --- | --- | --- | --- |
| 3000 | 4000 | 7000 | 5900 | 4900 |
| 3000 | 2000 | 5000 | 4500 | 5500 |
| 0 | 0 | 0 | 4000 | 4000 |
| 4000 | 0 | 4000 | 2800 | 6800 |
| 4000 | 4000 | 8000 | 5600 | 5600 |

**How much do you contribute to the joint account?**

______________ Ft

If the payoff at the end of the experiment occurs according to this situation, then the selected participants will receive the final earnings.

(In the original answer sheets, each situation was presented on a new sheet. To save space here, we present the situations compressed.)

# Situation 2

Suppose that you receive 3000 Ft and you can use part of that amount to place a bet (between 0 and 3000 Ft) on a colour in the next gamble.

There is a bag that contains 10 black and 10 red balls. We will draw one. If the colour of the ball drawn coincides with your bet, then we double the amount of your bet.

**How much would you bet?**

**Amount of the bet:**  __________ Ft

**Selected colour:**  __________

If the payoff at the end of the experiment occurs according to this situation, then the selected participants will receive the amount of money not used for the bet (3000-bet) and the money won on the bet.

# Situation 3

This situation is quite similar to the previous one, but there is an important difference.

Suppose that you receive 3000 Ft and you can use part of that amount to place a bet (between 0 and 3000 Ft) on a colour in the next gamble.

There is a bag with black and red balls, but it is unknown how many of the balls are black / red. We will draw a ball and if the colour of the ball drawn coincides with your bet, then we double the amount of your bet.

**How much would you bet?**

**Amount of the bet:**  __________ Ft

**Selected colour:**  __________

If the payoff at the end of the experiment occurs according to this situation, then the selected participants will receive the amount of money not used for the bet (3000-bet) and the money won on the bet.

# Optional task

This task is optional

If you find the solution of the following maze and you will be selected, then we give you an additional 300 Ft on top of the other earnings.


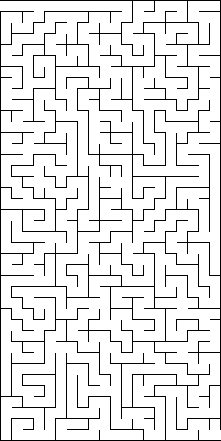


# Situation 4

In this situation you have to choose between earnings today and earnings in the future.

You may decide to have 3500 Ft in a week or a lower amount today. Choose according to if you prefer a given amount today or 3500 Ft in a week. Mark one of the possibilities in each row.


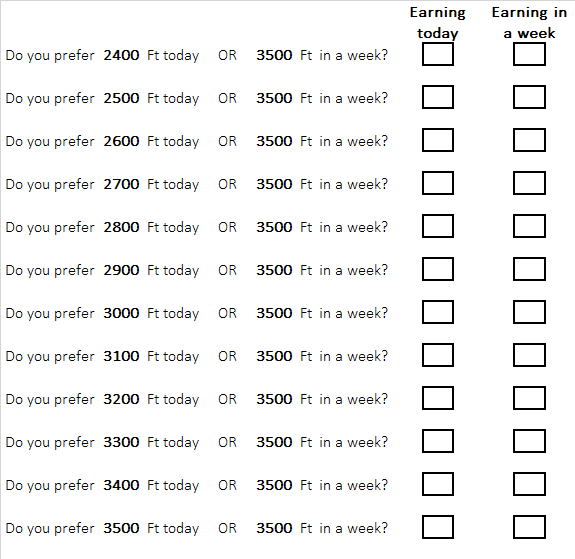


If the payoff at the end of the experiment occurs according to this situation, then first we select which of the above question will determine the earning. The selected participant will receive her / his earning according to her / his choice for the selected question and the earning will be paid immediately or in a week. If according to her / his choice the earning is to be paid in a week, then we put the corresponding amount of money in a sealed envelope that she / he will receive next week in this class from the professor. If this arrangement is not convenient for her / him, then she / he can collect the money at any time after the class in a week at the secretary of the Department of Economics (E3.59).

# Situation 5

This situation is very similar to the previous one, but now you have to choose between amounts of money to be received in two or three weeks.

Choose according to if you prefer a given amount in two weeks or 3500 Ft in three weeks. Mark one of the possibilities in each row.

**
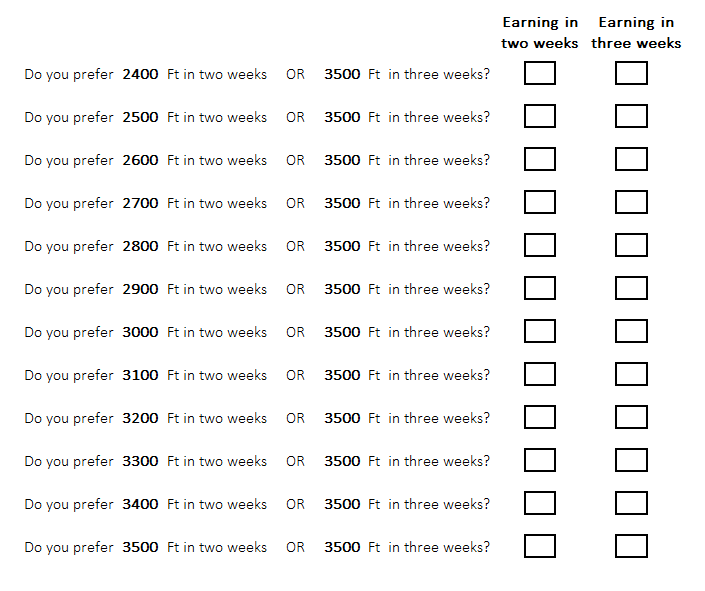
**

If the payoff at the end of the experiment occurs according to this situation, then first we select which of the above question will determine the earning. The selected participant will receive her / his earning according to her / his choice for the selected question and the earning will be paid according to her / his choice. We put the corresponding amount of money in a sealed envelope that she / he will receive in this class from the professor on the corresponding week. If this arrengement is not convenient for her / him, then she / he can collect the money at any time after that class at the secretary of the Department of Economics (E3.59).

# Quiz

Next you find a quiz of 8 questions that is necessary for situation 6. Please answer the questions before proceeding. You are not allowed to use any help. After completing the quiz, please answer the following questions that will determine your earnings for this situation. (We will tell the correct answers of the quiz at the end of the experiment.)

1. 4 cats eat 4 cans of cat food in 4 days. How long does it take for 40 cats to eat 40 cans? _______
2. In a pond, algae begin to expand and each day it doubles the surface covered. If it covers the entire pond in 10 days, how long does it take to cover half of the pond? _______
3. A flashlight with battery costs $3.3. The flashlight costs $3 more than the battery. How much does the battery cost? _______
4. How many neighbouring country does Switzerland have? _______
5. How many carbon atoms does a glucose molecule have? _______
6. How many verses does an elegiac couplet have? _______
7. How many members should the Hungarian Constitutional Court have at least? _______
8. How many seasons did the series „Friends” have? _______

**What do you think, how many of the questions did you answer correctly?**

__________

# Situation 6

Choose a compensation scheme for your answers given in the quiz. You may choose to be paid according to the number of correct answers. In this case, you will receive 250 Ft for each correct answer. Alternatively, you may choose to be paired randomly with two other participants and if the number of correct answers that you gave is higher than any of the other two participants, then you will receive 4500 Ft. In case of a tie (with the participant that had more correct answers), you will receive 2500 Ft. If the number of correct answers that you gave is less than the number of correct answers of the participant that scored better, then you receive nothing.

**What do you choose?** (Underline your choice)

Being paid according to the number of correct answers

OR

Being paid after comparing with two randomly chosen participants

If the payoff at the end of the experiment occurs according to this situation, then the selected participant receives the amount according to the chosen scheme. She / he receives an extra 250 Ft if she / he correctly guessed the number of correct answers.

# Questionnaire

Gender:

Year of birth:

Educational attainment

Vocational school

High school

BA degree

MA degree

other: _________________

**We thank for your cooperation! When you finish, please let it know the experimenters so the he can collect the answer sheets. Remember to keep one of the number tags.**
