# Supplementary material for: Which preferences associate with school performance?—Lessons from an exploratory study with university students
Source: PLoS One. 2018 Feb 16;13(2):e0190163. doi: 10.1371/journal.pone.0190163 (PMC5815576; doi:10.1371/journal.pone.0190163)
Supplement: S2 Appendix — (DOCX) [file pone.0190163.s002.docx]

# Appendix B – Measurement validation: Dean and Ortoleva [76]

Dean and Ortoleva [76] have measured in a single experiment with 190 subjects many of the preferences that we have investigated also.

Dean and Ortoleva [76] measure time preferences (they call them discount rates) as we do, that is using lists of choices that involve different amounts of money at different points in time. However, the amounts of money and the time horizons are different. Related to time preferences, they also measure present bias that captures the idea that individuals tend to exhibit higher discount rates when the payment is available immediately.

Dean and Ortoleva [76] measure risk aversion by eliciting certainty equivalents in three 50/50 lotteries and the difference between the expected value of the lottery and the certainty equivalent is taken as risk aversion. We use also a 50/50 lottery, but we measure risk aversion as the amount of money that the individuals are willing to bet on that lottery. Dean and Ortoleva [76] also measure uncertainty aversion (that they call ambiguity aversion) in the same vein as we do.

Dean and Ortoleva [76] do not measure social preferences as we do, but they use the trust game to capture social concerns. Given the difference in the measures, no direct comparison can be made. Dean and Ortoleva [76] do not measure competitive preferences.

They gauge cognitive skills with Raven’s Matrices, a standard measure of perceptual reasoning. Similarly to us, they also assess overconfidence (they call it overestimation) as the difference between the number of questions that they think they got right in the test and the number of actual performance in the test.
